# Genome Sequences of *Gordonia* Bacteriophages Jodelie19, BlingBling, and Burnsey

**DOI:** 10.1128/MRA.01280-20

**Published:** 2021-02-04

**Authors:** D’Andrew L. Harrington, Hannah R. Barten, Elizabeth I. Audannio, Fredrick A. Bragg, Karla A. Garcia, Katelyn M. Niswonger, John R. MacAllister, Ariana M. McCarroll, Devon M. O’Sullivan, Silvio E. Torres, Craig D. Stanley, Dariush Yalzadeh, Kendra W. Kimberley, Chelsey C. McKenna, James R. Theoret, Earl J. Yoon, Erin J. Windsor

**Affiliations:** a Department of Biological Sciences, College of Southern Nevada, Las Vegas, Nevada, USA; Queens College

## Abstract

Jodelie19, BlingBling, and Burnsey are bacteriophages identified using host bacteria of the genus *Gordonia*. Jodelie19 is a lytic phage found in Gordonia rubripertincta NRRL B-16540. The temperate phage BlingBling and lytic phage Burnsey were both isolated using the host bacterium Gordonia terrae 3612.

## ANNOUNCEMENT

**FIG 1:**
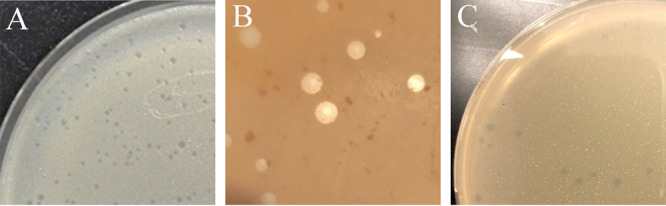
Images of plaques of bacteriophages Jodelie19 (A), BlingBling (B), and Burnsey (C). Jodelie19 and BlingBling show small cloudy plaques. Burnsey shows small clear plaques.

All three bacteriophages were collected as part of the Science Education Alliance-Phage Hunters Advancing Genomics and Evolutionary Science (SEA-PHAGES) program (1). The SEA-PHAGES program isolates bacteriophages from *Actinobacteria*, like *Gordonia* spp., due to their ability to serve as human pathogens. *Siphoviridae* Jodelie19 was collected from damp soil located by a pond in Las Vegas (global positioning system [GPS] coordinates, 36.2872N, 115.177W). Jodelie19 was discovered from direct isolation and displayed cloudy plaques 1 mm in diameter (Fig. 1A). *Siphoviridae* BlingBling was discovered by direct isolation from the University of Pittsburgh from dry soil and produced turbid plaques (GPS coordinates, 40.443889N, 79.950639W) (Fig. 1B). *Siphoviridae* Burnsey was discovered by enriched isolation from Durham Technical Community College at Booker Creek Trail from wet soil and produced small clear plaques (GPS coordinates, 35.947415N, 79.026757W) (Fig. 1C). The bacteria were grown at 30°C in peptone yeast calcium agar (PYCa) medium in a shaking incubator at 200 rpm for 48 h prior to use for plaque assays. All protocols for bacteriophage isolation, purification, and DNA extraction are published in the SEA-PHAGES Phage Discovery Manual (2). DNA was isolated using the Promega DNA Wizard cleanup kit. Sequencing of Jodelie19, BlingBling, and Burnsey was completed by the Pittsburgh Bacteriophage Institute using an Illumina MiSeq instrument. Sequencing libraries were generated from extracted genomic DNA using New England BioLabs (NEB) Ultra II library kits, per the manufacturer’s instructions. Sequencing provided 150-base pair single-end reads. There was 3,357× coverage for BlingBling with 1,156,314 reads. There were 833,697 reads for Burnsey with 23× coverage and 790,458 reads for Jodelie19 with 1,814× coverage. Raw reads were assembled with Newbler v2.9 with default settings and in each case produced a single phage contig. For quality control, these contigs were analyzed using the default settings of Consed v29 (http://www.phrap.org/consed/consed.html) to verify accuracy and complete genome circularization and to determine genomic termini as previously described (3). Consed v29 confirmed that all three bacteriophages have 3′ sticky overhangs. Jodelie19 has 62,331 bp and a GC content of 51.4%. Burnsey has 46,185 bp with a GC content of 60.3%, and BlingBling has 48,746 bp with a GC content of 67.1%.

Annotations of Jodelie19, BlingBling, and Burnsey were completed with the following programs: DNA Master v5.23.2 (http://cobamide2.bio.pitt.edu/computer.htm), Starterator v1.2 (https://github.com/SEA-PHAGES/starterator), Phamerator (phamerator.org) (4), PhagesDB BLAST (phagesdb.org/blast) (5), NCBI BLAST (6), PECAAN (https://blog.kbrinsgd.org/), GeneMark v2.5p (7), Glimmer 3.02 (8), ARAGORN (v1.1 and v1.2.38) (9), HHPRED (v3.2.0) (10), tRNAscan-SE 2.0 (11), TMHMM (v2.0) (12), and SOSUI (v1.11) (13).

Putative functions were predicted for 23 of 91 of Jodelie19’s genes. Bioinformatic analysis of BlingBling’s 76 genes allowed for a putative function assignment for 35 genes and 30 genes out of 70 genes for Burnsey. *Gordonia* bacteriophages are clustered together if they share at least 35% of their genes (14). Jodelie19 aligns with the cluster DJ phages, BlingBling to CV, and Burnsey to CT. Bacteriophages in the cluster CV tend to be temperate (5). BlingBling was confirmed to be temperate during bioinformatic analysis with the presence of excise, immunity repressor, and tyrosine integrase genes.

### Data availability.

Jodelie19 GenBank and SRA accession numbers are MT498052 and SRR12707452, respectively. BlingBling GenBank and SRA accession numbers are MT553336 and SRR12707454, respectively. Burnsey GenBank and SRA accession numbers are MT889378 and SRR12707453, respectively.
